# MicroRNA 199a-5p induces apoptosis by targeting JunB

**DOI:** 10.1038/s41598-018-24932-9

**Published:** 2018-04-27

**Authors:** Mengjie Yan, Sibao Yang, Fanbo Meng, Zhihui Zhao, Zhisen Tian, Ping Yang

**Affiliations:** 1 Department of Internal Medicine and Cardiology, China-Japan Union Hospital of Jilin University, Changchun, 130033 China; 2 College of Animal Science and Veterinary Medicine, Jilin University, Changchun, 130062 China; 3 Department of orthopedics, China-Japan Union Hospital of Jilin University, Changchun, 130033 China

## Abstract

MicroRNAs participate in a variety of physiological and pathophysiological processes in various organs including the heart. Our previous work revealed that the level of miR-199a-5p was significantly higher in failing hearts than in control hearts. However, whether it is associated with the progression of heart failure (HF) and mediates cardiomyocyte apoptosis remained unclear. In the present study, we used various biochemical and molecular biological approaches to investigate the changes in miR-199a-5p levels in failing hearts in a rat model induced by acute myocardial infarction. We found that miR-199a-5p levels in the heart increased with the progression of HF, and overexpression of miR-199a-5p significantly increased apoptosis in untreated H9C2 cells and potentiated angiotensin II-induced apoptosis. Thus, our results indicate that miR-199a-5p is involved in the progression of HF and mediates cardiomyocyte apoptosis. We also confirmed that JunB, a member of the activator protein-1 transcription factor family, is one of direct targets of miR-199a-5p via a dual-luciferase reporter assay and mutagenesis on the 3′ untranslated region of the JunB gene. Consistent with the above findings, overexpression of JunB in H9c2 cells suppressed cell apoptosis. Based on our findings, miR-199a-5p induces apoptosis by targeting JunB.

## Introduction

Apoptosis, an energy-dependent programmed cell death, is an important biological process for tissue renewal and organ development *in vivo* that also can induce pathophysiological changes^1^. For instance, apoptosis in cardiac myocytes may lead to irreversible myocardial injury and therefore is involved in a variety of pathological processes such as pathological ventricular remodeling and myocardial infarction^2^, thus contributing to the pathogenesis of various cardiovascular diseases including heart failure (HF). It is therefore widely believed that suppression of apoptosis may ameliorate pathological changes in heart tissues and benefit cardiac function in a variety of cardiac diseases.

MicroRNAs (miRNAs) are a sort of short non-coding RNAs that regulate physiological and pathological processes through binding to the 3′ untranslated region (UTR) of the target gene mRNA and subsequently causing target gene degradation^3^. Many miRNAs are released into circulation during certain disease processes and are considered potential biomarkers for those diseases, including HF^4^. For instance, miR-208 is a heart-specific miRNA that is released into the blood stream from injured cardiomyocytes^3^. The level of circulating miR-21 is associated with cardiac hypertrophy, and it protected myocytes from H_2_O_2_-induced injury by targeting PDCD4^5^. In addition, miRNAs have been shown to take part in myocyte apoptosis. For example, miR-101 and miR-29b both target Mcl-1 to prevent apoptosis^6,7^, while miR-499a impairs myocyte survival by repressing the expression of histone deacetylases^8^. In addition, miR-34a induces apoptosis by down-regulating the expression of SIRT1 and stimulating the p53 pathway^9^, while miR-16 was implicated in cell proliferation and apoptosis through the targeting of Bcl-2^10^. All these findings point to the potential role of miRNAs in cardiac homeostasis.

A previous study showed differential expression of miRNAs in failing hearts of a rat model compared with control hearts, including 79 upregulated miRNAs and 28 downregulated miRNAs^11^. Among those upregulated miRNAs, miR-199a-5p expression was 3.4-fold higher in failing hearts compared with control hearts^11^. miR-RNA-199, first cloned from an osteoblast sarcoma cell line (Saos-2)^12^, is highly conserved in human and mouse. Two mature miRNA-199s, miR-199a-5p (from the 5′ arm) and miR-199a-3p (from the 3′ arm), are processed from the same stem loop precursor RNA. Recent studies indicated a potential role for miR-199a-5p in cardiac hypertrophy induced by G protein coupled receptor (GPCR) agonists, such as phenylephrine (PE) and angiotensin II (Ang II)^13,14^. Mechanistically, miR-199a-5p regulates cell proliferation, motility, and angiogenesis by directly and indirectly targeting caveolin-1 (Cav-1)^15–17^. However, whether miR-199a-5p can directly mediate cardiomyocyte apoptosis is poorly understood.

It is well documented that octapeptide Ang II plays an important role in cardiac homeostasis; increased activity of Ang II promotes cardiomyocyte apoptosis^18^. Mechanistically, Ang II induces cardiomyocyte apoptosis at least through the following mechanisms: (1) AT1 receptor-dependent increase of 20-HETE and CYP4A enzyme promotes apoptosis through the impairment of mitochondria^19^; (2) the ligand binding of the Ang ll receptor on the cell membrane transfers the death signal to the nucleus and activates Ca^2+^-dependent DNase I, causing DNA fragmentation induced by nuclear cleavage, cell shrinkage, and apoptotic body formation^2^; (3) Ang II activates the classical p53-dependent apoptotic pathway (1980)^20^; and (4) Ang II stimulates transforming growth factor beta (TGF-β) expression and induces autophagy through SMAD pathway (7).

In the present study, we aimed to compare the levels of miR-199a-5p and its predicted target gene JunB between an end-stage HF group and a control group and to investigate whether miR-199a-5p and JunB are involved in cardiomyocyte apoptosis *in vitro*.

## Results

### End-stage HF mice exhibited increased expression of miR-199a-5p and decreased expression of JunB in hearts

**Figure 1:**
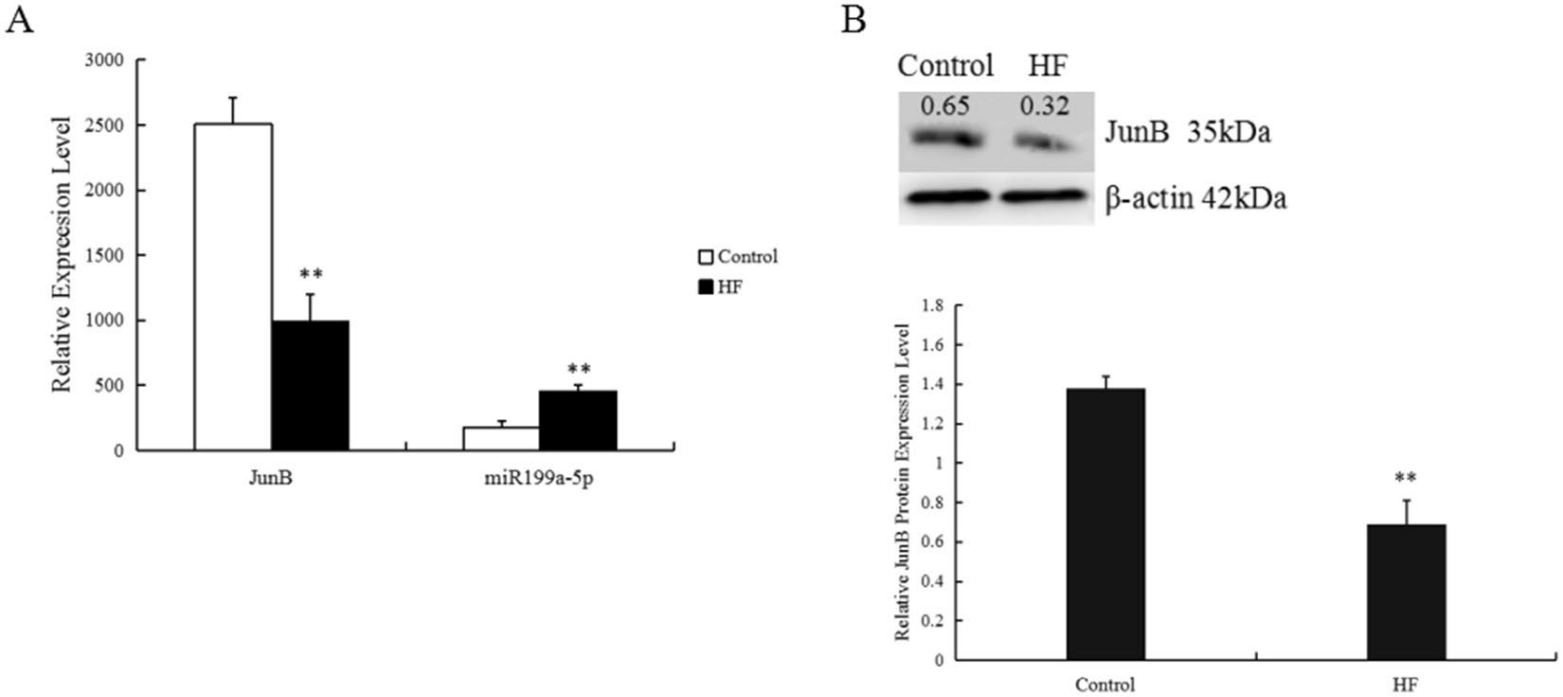
MI mice exhibited increased expression of miR-199a-5p and decreased expression of JunB in heart. MiR-199a-5p levels in the hearts of the HF and sham operation groups were evaluated by RT-qPCR by using oligos specifically designed to target mir-199a-5p. JunB levels were evaluated using RT-qPCR and Western blotting. **P < 0.01 vs. sham. n = 3 per group.

RT-qPCR and Western blotting were performed on RNAs and proteins purified from cardiac tissues collected from rats of the sham and HF groups. As shown in Fig. 1, miR-199a-5p levels were significantly higher in the heart tissues of end-stage HF mice compared with in those of the sham group. We searched the targets of miR-199a-5p through a common database and screened several genes related to apoptosis, cell growth, and cell cycle. Among these predicted genes, we found that the mRNA and protein levels of JunB were significantly lower in the HF mice heart tissues compared with those of the control group (Fig. 1). We speculated that a relationship may exist between miR-199a-5p and JunB, and eventually focused on Jun B proto-oncogene (JunB) in our further research.

### A 100-nM dose of Ang II successfully induced apoptosis of myocardial cells *in vitro*

**Figure 2:**
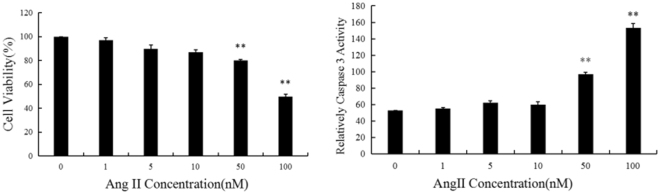
Ang II-induced apoptosis of H9C2 cells. Cultured H9C2 cells were stimulated with different concentrations of Ang II as indicated. The optimal working concentration was evaluated by MTS and caspase-3 activity assays. **P < 0.01 vs. vehicle (0 group). n = 3 per group.

We next tested the optimal working concentration of Ang II for the induction of apoptosis in H9C2 cells, a rat cardiac cell line. H9C2 cells seeded in 96-well plates were stimulated with different Ang II concentration for 24 h as indicated in Fig. 2. The 100 nM dose of Ang II most significantly inhibited the viability in H9C2 cells as evaluated by the MTS method and increased caspase-3 activity (**P < 0.01 vs 0 nM Ang II).

### Increasing Ang II concentration elevated miR-199a-5p expression *in vitro*

**Figure 3:**
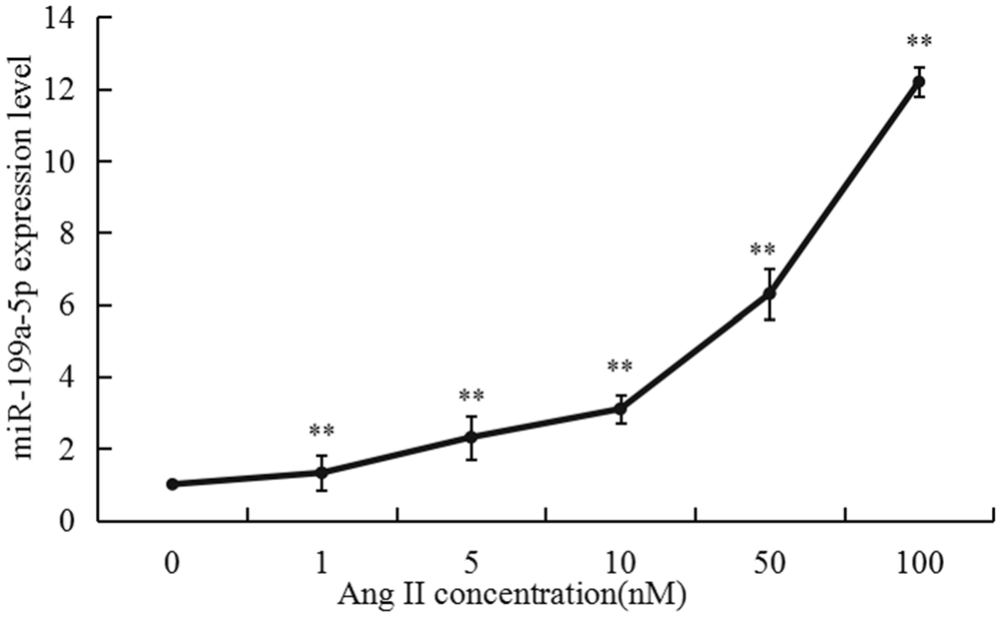
Stimulation with Ang II increased concentration caused miR-199a-5p levels in a dose dependent manner in H9C2 cells. Different concentrations of Ang II (1–100 nM) were used to stimulate H9C2 cells as described in the Materials and Methods. The miR-199a-5p expression level was determined by quantitative RT-PCR. **P < 0.01, vs. NC (0 nM group).

Ang II stimulation of H9C2 cells for 24 h increased miR-199a-5p levels in a dose-dependent manner, with 1 nM Ang II treatment significantly increasing miR-199a-5p levels compared with 0 nM group (**P < 0.01, Fig. 3).

### miR-199a-5p promoted apoptosis in H9C2 cells

**Figure 4:**
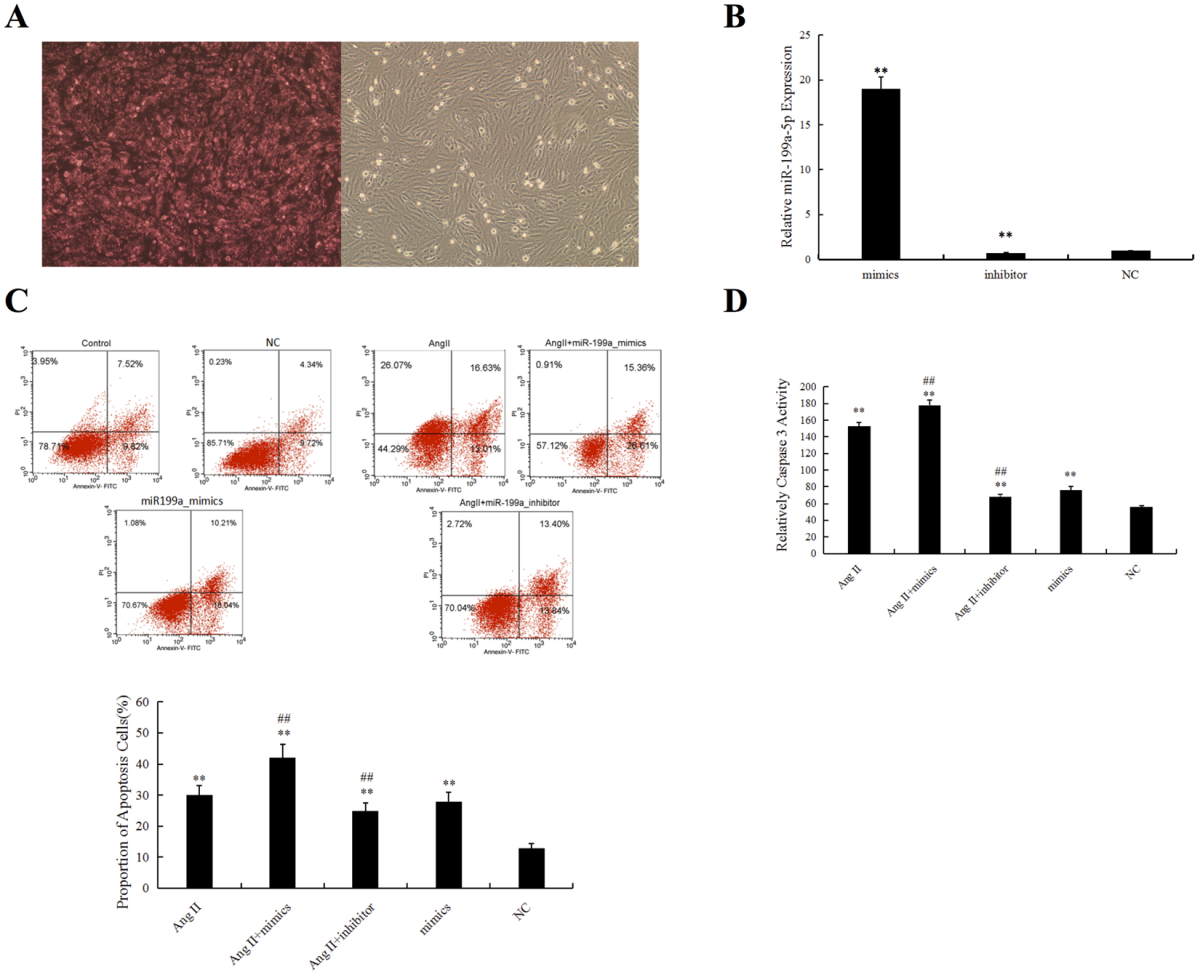
MiR-199a-5p exacerbated Ang II-induced apoptosis in H9C2 cells. (**A**) Evaluation of transfection efficiency in H9C2 cells transfected with fluorescently labeled miR-199a-5p mimics. Transfected cells stained red. (**B**) Evaluation of miR-199a-5p expression in H9C2 cells transfected with (NC) miR-199a-5p mimics and a miR-199a-5p inhibitor by RT-qPCR. **P < 0.01 vs. NC. (**C**) Involvement of miR-199a-5p in Ang II-induced apoptosis in H9C2 cells. Flow cytometry was used to identify apoptotic cells (red) stained with anti-annexin V and propidium iodide. The lower panel shows the summary and statistical analysis of three independent analyses of the upper panel. **P < 0.01 vs. NC. ^##^P < 0.01 vs. Ang II group. (**D**) A caspase-3 activity assay was used to evaluate cell apoptosis among the different groups. **P < 0.01 vs. NC. ^##^P < 0.01 vs. Ang II group.

To further explore the effect of miR-199a-5p on apoptosis, we overexpressed or inhibited miR-199a-5p in H9C2 cells in the presence of Ang II stimulation. We first evaluated the transfection efficiency of fluorescently labeled miR-199a-5p mimics, inhibitors, and negative controls in H9C2 cells by fluorescence microscopy, and 80–90% of H9C2 cells were stained red (Fig. 4A). We also used real-time PCR to examine the transfection efficiency. The expression level in the overexpression group was significantly higher than that in the normal control group (P < 0.01), and miR-199a-5p expression was suppressed in the inhibitor group (P < 0.01) compared with the normal control group (Fig. 4B).

As expected, compared with that in the control group, flow cytometry (FCM) showed that the percentage of apoptotic cells was significantly increased by Ang II stimulation, which was further enhanced by the overexpression of miR-199a-5p but suppressed by the inhibition of miR-199a-5p (Fig. 4C). The caspase-3 activity of myocytes measured in different groups was consistent with the FCM data (Fig. 4D). Thus, we argue that miR-199a-5p is involved in mediating apoptosis of myocardial cells *in vitro*.

### MiR-199a-5p activated proapoptotic genes

**Figure 5:**
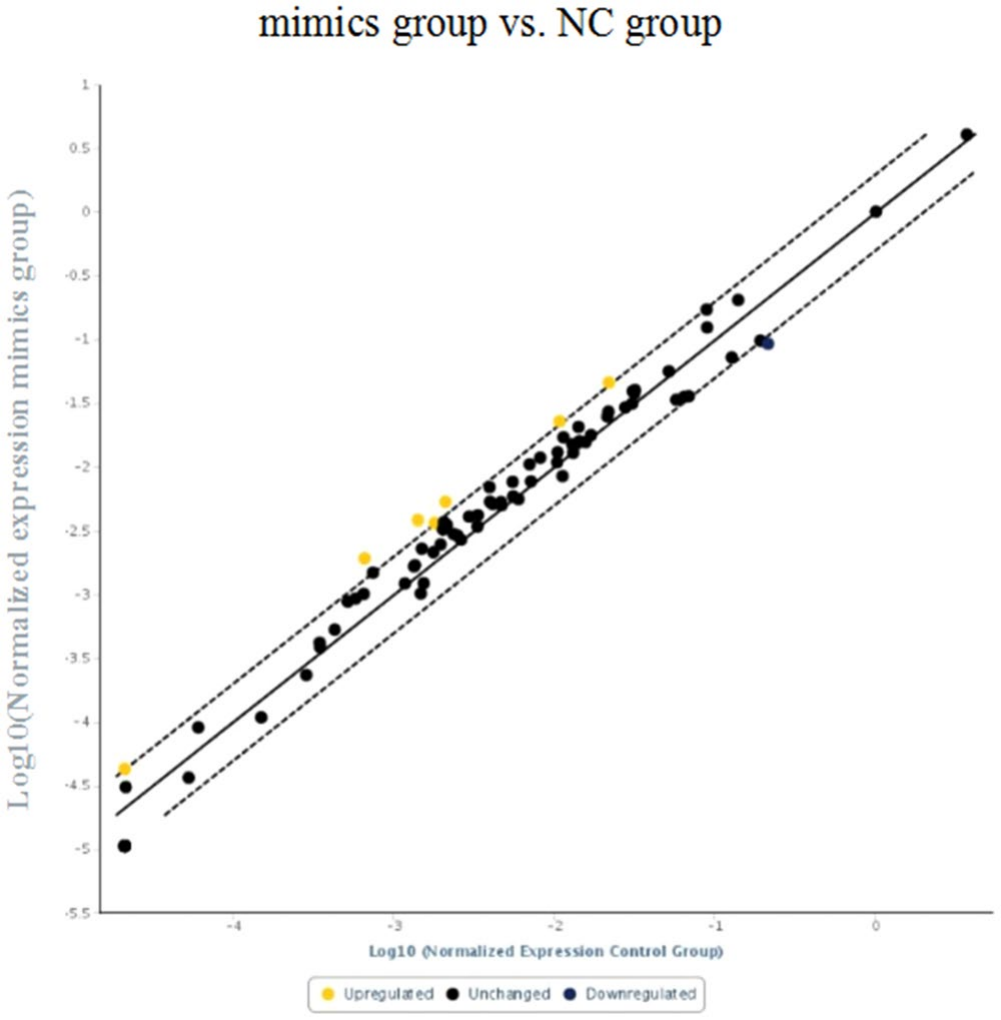
Rat Apoptosis RT² Profiler™ PCR Array analysis. Expression levels of seven pro-apoptotic genes were increased significantly upon miR-199a-5p overexpression.

Since miR-199a-5p induced cell apoptosis, we next investigated whether genes related to the apoptotic pathway are differentially regulated by miR-199a-5p overexpression using Rat Apoptosis RT² Profiler™ PCR Array analysis. Of 84 apoptotic pathway-related genes examined, 7 showed significant differences in expression between the miR-199a-5p overexpression group and the control group (Fig. 5 and Table 1). Specifically, the results revealed significantly increased expression (fold-change ≥2.0) of 7 pro-apoptosis genes (Casp8, Casp4, Casp12, Abl1, Casp1, Casp6, and Tnfsf12) as well as increased expression of other pro-apoptosis genes like Casp3, Casp7, and Apaf1 (fold-change ≥1.0, ≤2.0). In addition, the expression of anti-apoptotic genes such as Bcl2, Bcl2l1, and Bcl2l11 was decreased to varying degrees (fold-change ≥1.0, ≤2.0). Therefore, we conclude that miR-199a-5p promotes apoptosis by enhancing the expression of pro-apoptotic genes and suppressing the expression of anti-apoptotic genes.

**Table 1:** Seven upregulated genes in MI group compared to control group.

Genes	Casp8	Casp4	Casp12	Abl1	Casp1	Casp6	Tnfsf12
Fold change	2.93	2.71	2.53	2.11	2.03	2.01	2.00

All P < 0.01.

### Apoptotic cells exhibited increased expression of miR-199a-5p and decreased expression of JunB

**Figure 6:**
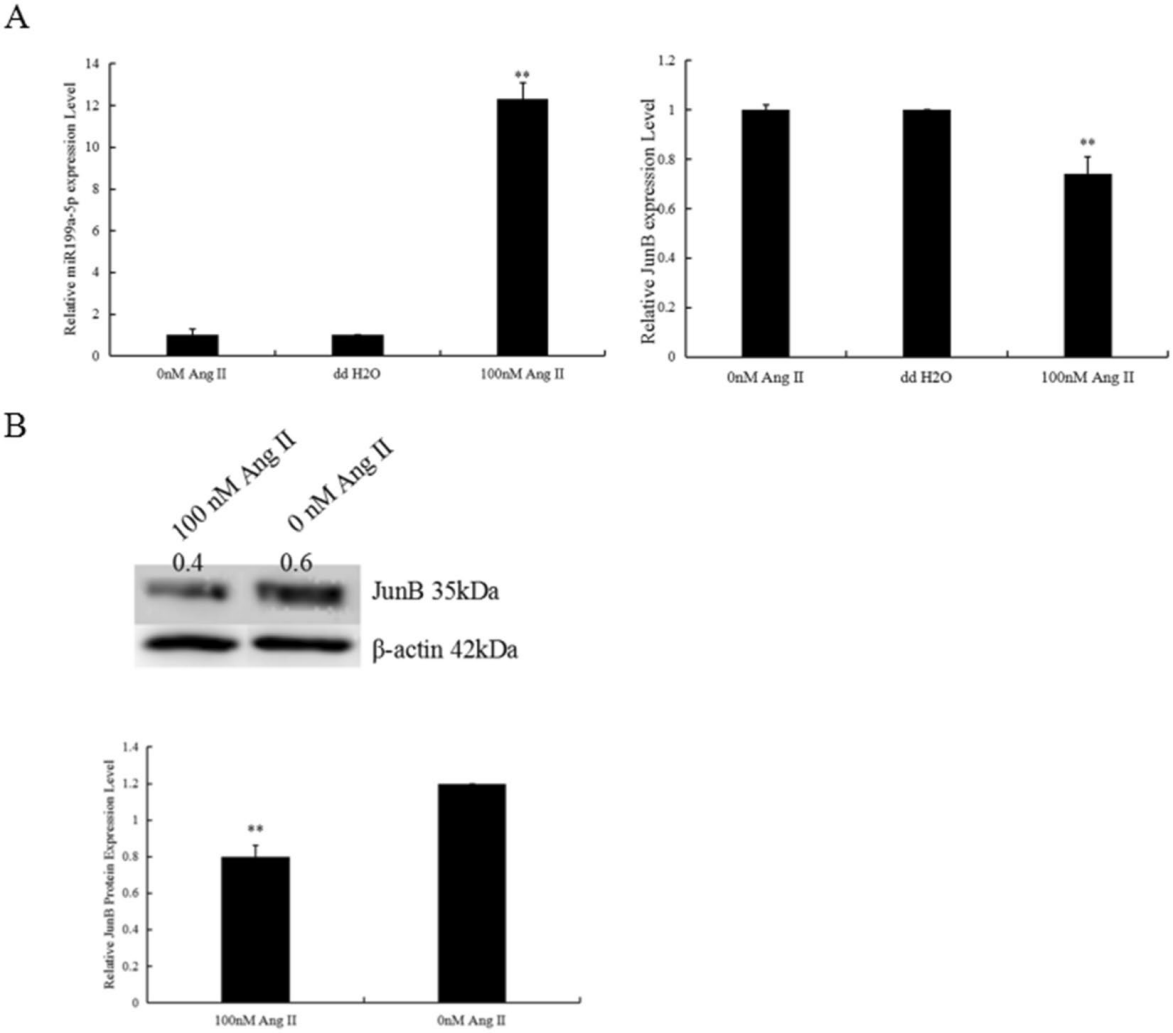
MiR-199a-5p expression increased and JunB expression decreased in Ang II-treated myocardial cells. Evaluation of miR-199a-5p and JunB levels by RT-qPCR and Western blotting. **P < 0.01 vs. vehicle (0 group). n = 3 per group.

After exposure of H9C2 cells to 100 nM Ang II for 24 h, miR-199a-5p expression was significantly increased and JunB expression was decreased (Fig. 6).

### Identification of JunB as a target of miR-199a-5p

**Figure 7:**
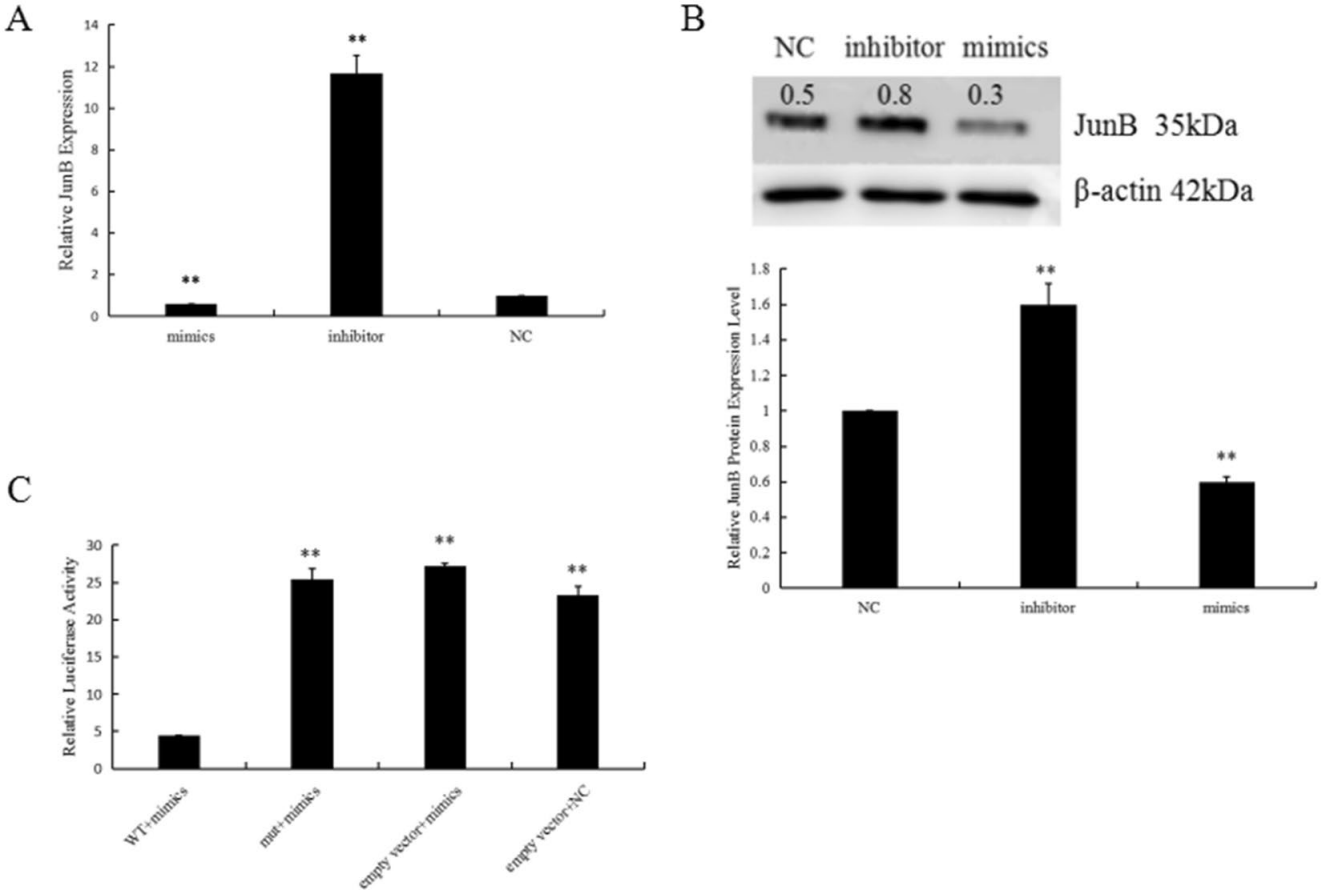
JunB was identified as a direct target of miR-199a-5p. JunB expression was mediated by the miR-199a-5p levels in H9C2 cells transfected with mimics, inhibitor and NC as evaluated by RT-qPCR (**A**) and Western blot (**B**) analyses. **P < 0.01 vs NC. β-actin was used as an internal control (**B**). (**C**) miR-199a-5p targeted the 3′UTR of JunB. Reporter assays were carried out as described in the Materials and Methods. Data were compiled from three independent experiments.

We examined the expression of JunB in H9C2 cells transfected with normal control, mimics, and inhibitors of miR-199a-5p by both RT-qPCR and Western blot. As shown in Fig. 7A, the mimics significantly lowered JunB levels compared with the inhibitor or normal control. Consistent with this, Western blot analysis also revealed that JunB expression was greatly reduced or increased, in mimic- or inhibitor-transfected cells, respectively (Fig. 7B). Thus, we argue that miR-199a-5p mediates JunB expression.

MiRNAs inhibit the expression of their target genes by binding to the seed sequence in the 3′UTR of the gene, and JunB was predicted to be a potential target gene for miR-199a-5p through bioinformatics analysis. To verify whether JunB is the target of miR-199a-5p, we cloned the wild-type (WT) and mutant miR-199a-5p binding sites on the 3′UTR of the JunB gene into the pmiR-RB-REPORT vector. As expected, pmiR-RB-REPORT™-WT was significantly inhibited by miR-199a-5p mimics, but pmiR-RB-REPORT™-mut was not (Fig. 7C). Thus, we conclude that miR-199a-5p inhibits Jun B expression by targeting the binding sequence located in its 3′ UTR.

### Overexpression of JunB in H9c2 cells reduced cell apoptosis

**Figure 8:**
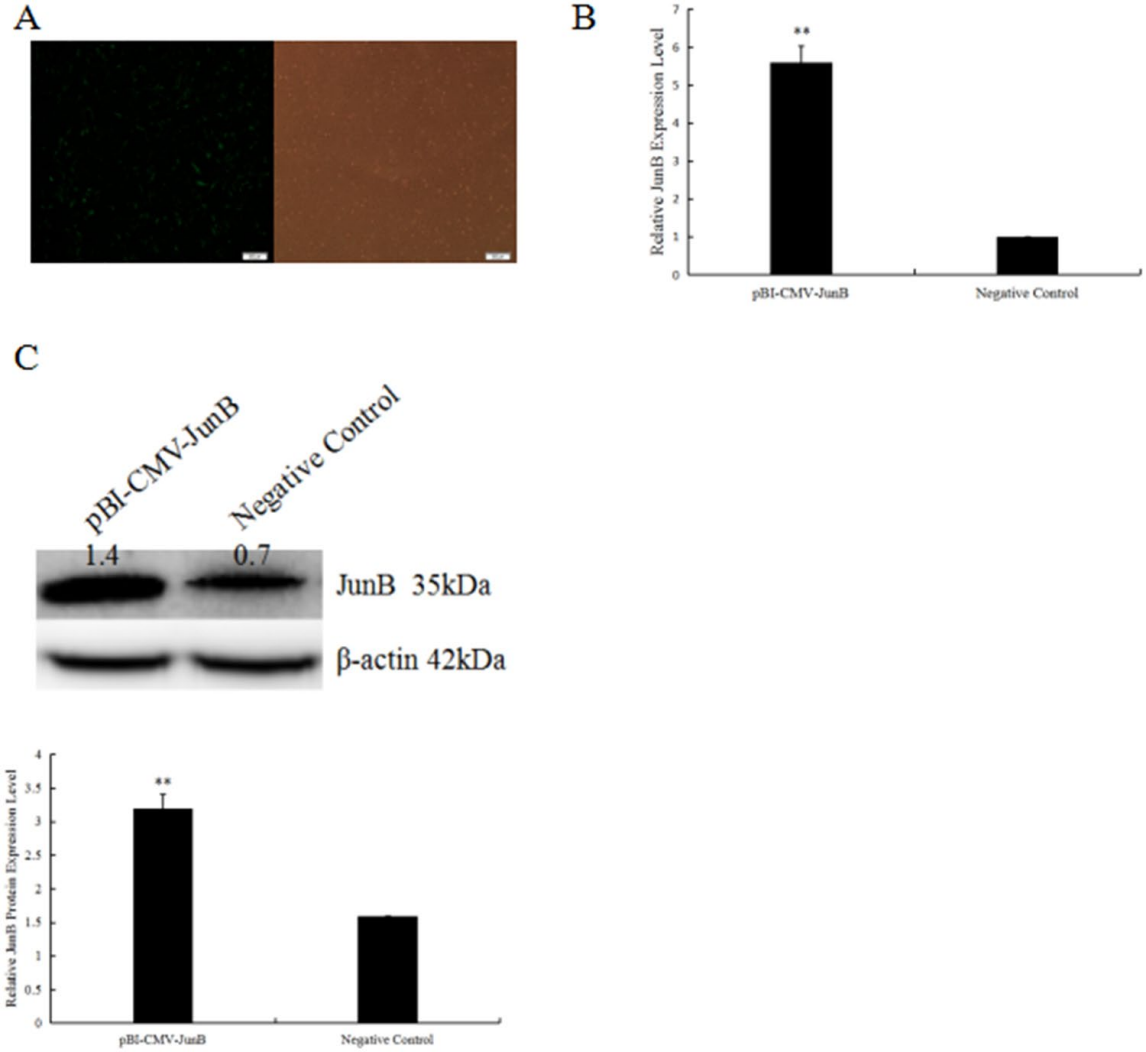
H9C2 cells were successfully transfected with PBI-CMV3-JunB vectors. (**A**) Evaluation of transfection efficiency of pBI-CMV3-JunB, which expressed a ZsGreen green fluorescent protein, in H9C2 cells. Transfected cells stained green. (**B**&**C**) Evaluation of JunB expression in H9C2 cells transfected with pBI-CMV3-JunB and NC by RT-qPCR and Western blotting. **P < 0.01 vs. NC.

**Figure 9:**
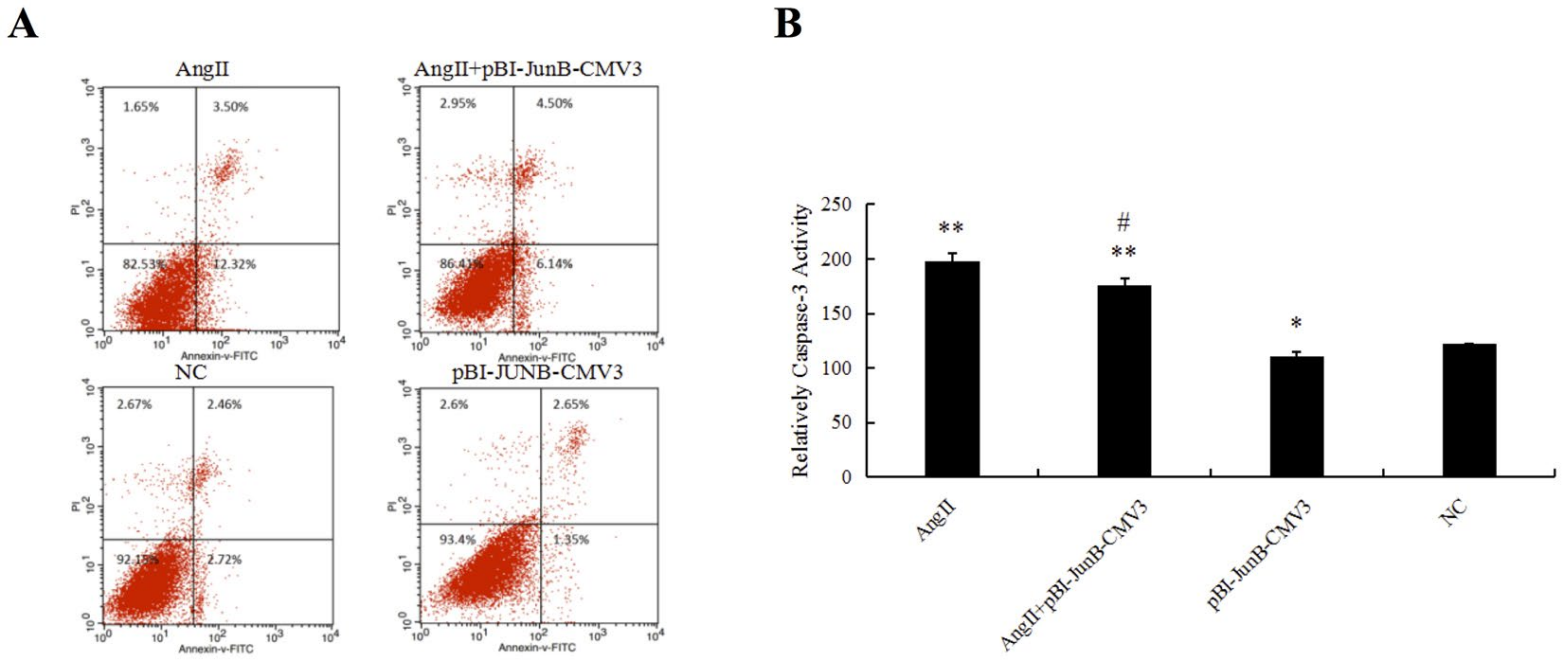
JunB functioned as a protective factor against myocardial cell apoptosis. (**A**) Flow cytometry was used to identify apoptotic cells (red) stained with anti-annexin V and propidium iodide. (**B**) A caspase-3 activity assay kit was used to evaluate cell apoptosis among the different groups. **P < 0.01 vs. NC, *P < 0.05 vs. NC, ^#^P < 0.05 vs Ang II group.

To explore the role of JunB in myocardial cell apoptosis, we constructed a pBI-CMV3 vector that carried GFP for continuous overexpression of JunB in cells. As shown in Fig. 8, JunB was successfully expressed as evaluated by the green fluorescence of the pBI-CMV3 vector, real-time PCR, and Western blotting. We found that JunB reduced apoptosis and caspase-3 activity induced by Ang II (Fig. 9A,B). Thus, we suggest that JunB functions as a protective factor against cell apoptosis.

## Discussion

In the present study, we used heart tissues of a rat HF model and sham operation group to explore mir199a-5p and JunB expression patterns and the roles they play in myocardial cell apoptosis. Our major findings are: (1) cardiac miR-199a-5p expression was increased in the HF heart tissues compared with the sham-operation group, and JunB expression was relatively lower in the HF group; (2) mir-199a-5p expression increased and JunB expression decreased after stimulation with Ang II; (3) miR-199a-5p promoted apoptosis induced by Ang II stimulation; (4) Jun B was a direct target of miR-199a-5p; and 5) JunB played a protective role in myocardial apoptosis.

A recent study indicated that the serum miR-199a-5p level is a potential biomarker in osteosarcoma patients^21^. It would be of great interest to explore if circulating mir-199a-5p levels in AMI patients are also increased and can also serve as a valuable biomarker for myocardial cell injury. In addition, our findings support the premise that miR-199a-5p is a potential therapeutic target for treatment of MI in the clinic, as revealed by another study showing that one of the mechanisms by which atorvastatin prevents ischemia-reperfusion-induced cardiac injury is suppression of miR-199a-5p expression^22^.

Another interesting finding from our study was that miR-199a-5p exhibited pro-apoptotic activity. The roles of miR-199a-5p in cell proliferation in both cancer and the heart have been well documented. For instance, miR-199a-5p promotes proliferation and migration of gastric cancer cells via targeting klotho^23^. In the cardiac setting, miR-199a-5p expression is elevated in hypertrophic hearts, and overexpression of miR-199a-5p induced cardiomyocyte proliferation and enlarged cell size^24,25^. However, whether miR-199a-5p affected cardiomyocyte apoptosis was not completely clear. In the present study, we treated cardiomyocytes with AngII and found that 100 nM AngII induced significant apoptosis. We also found that miR-199a-5p mimics not only promoted Ang II-induced apoptosis, but also induced apoptosis in H9C2 cells.

In this study, we provided evidence that JunB is a direct target of miR-199a-5p. JunB is a member of the activator protein–1 (AP-1) transcription factor family belonging to the transcription factor protein family comprising Jun (c-Jun, JunB and JunD) and Fos (c-Fos, FosB, Fra-1 and Fra-2)^26^. JunB binds to the cognitive binding sequence localized in the cis-regulatory domain of target genes and regulates a variety of biological processes including cell cycle, proliferation, and apoptosis. For instance, JunB was shown to inhibit proliferation of malignant mouse keratinocytes once overexpressed^27^, while it suppressed endoplasmic reticulum (ER) stress-induced cell apoptosis in pancreatic beta cells^28^. Consistently, in the vascular field, JunB was identified as a positive mediator of vasculogenesis and homeostasis but as a negative mediator of cell proliferation and apoptosis. For example, the JunB/miR-128/Foxo1 regulatory axis was shown to govern the development of lympathic vessels in zebrafish^29^, whereas endothelial-specific ablation of JunB mediated by Tie-2 cre resulted in embryonic lethality at E10.5 with embryos exhibiting abnormal vascular structures^30^. However, studies of the role of JunB in heart diseases have been limited. JunB accounts for HF originating from inflammatory cardiomyopathy as revealed in a transcriptome network analysis^31^, while morpholino-mediated JunB knockdown causes HF in zebrafish^32^. It was not clear, however, if JunB was implicated in apoptotic process. In the present study, we first showed that JunB expression was repressed by Ang II and downregulated in H9C2 cells once miR-199a-5p expression was increased. Based on our previous work (unpublished), JunB was downregulated in failing hearts and predicted to be a target for mir199a-5p. We also found that the expression of JunB was influenced by mir-199a-5p expression. Based on these findings together, we predicted that JunB may be a direct target for mir199a-5p according to the functional mechanism of the miRNA. We further used molecular approaches to identify JunB as a direct target of miR-199a-5p, as evidenced by the finding that an increase in miR-199a-5p activity suppressed the activity of the pmiR-RB-REPORT™-JunB-WT reporter but not the pmiR-RB-REPORT™-JunB-mut reporter. Given that miR-199a-5p promoted cardiomyocyte proliferation and apoptosis and that JunB repressed cell proliferation and apoptosis as observed in the cancer and vascular fields, it is highly likely that JunB is one of the molecules that mediates the downstream effects exerted by upregulated miR-199a-5p in HF, JunB is known to be involved in mediating cell apoptosis. In the present study, we found that JunB was a direct target of miR-199a-5p. Therefore, we believe that miR-199a-5p mediates apoptosis at least in part through regulating the activity of JunB. However, we did not rule out the possibility that miR-199a-5p may regulate the expression of those pro-apoptotic genes indirectly. Other mechanisms potentially underlying the regulation of AngII-induced apoptosis by miR-199a-5p remain to be elucidated. From JunB loss- and gain-of-function studies, we found that JunB protected H9c2 cells from apoptosis induced by Ang II.

In conclusion, we demonstrated in the present study that the expression of miR-199a-5p is upregulated in failing hearts of a rat AMI model. Also, miR-199a-5p promotes Ang II-induced cell apoptosis *in vitro* and directly suppresses the expression of its target JunB. In addition, JunB expression reduces cell apoptosis. Mechanistically, miR-199a-5p triggers apoptosis at least in part by suppressing JunB expression.

## Materials and Methods

### Generation of MI animal model, cardiac functional analysis and tissue collection

Fifty female Wistar rats were provided by the Basic Medical College of Jilin University. The left anterior descending artery of each rat was sutured to generate AMI model as described^11^. During the operation, two rats died from hyper-anesthesia, and the remaining rats were randomized to the AMI group (MI-10 weeks, MI-10W, n = 8) or sham-operation group (sham, n = 18). Breeding was terminated at 10 weeks after operation. Echocardiography was performed to determine the cardiac function of rats in each group as previously detailed^11^. The AMI group showed significantly lower FS and LVEF than the control group, as rats in the MI-10W group had the worst cardiac function compared with those in the other two AMI groups^11^. The serum BNP levels in rats of the three MI groups were significantly higher compared with that in the sham group, with rats in the MI-10W group having the highest circulating BNP levels^11^. These data confirmed the valid MI and heart failure rat model. The left ventricles of rats from each group were collected and snap-frozen in liquid nitrogen. All animals received humane care, and all experimental protocols were approved by the Animal Ethics Committee of Jilin University. All procedures involving animals were performed in accordance with the ethical standards of the institution or practice at which the studies were conducted.

### RNA extraction and reverse transcription quantitative polymerase chain reaction (RT-qPCR)

The myocardial tissues in the infarct area or the equivalent area collected from 3 rats of the AMI group and the sham group were stored in liquid nitrogen prior to total RNA extraction using TRIzol reagent (TaKaRa, Japan). The RNA concentration was measured with a NanoDrop 2000 spectrophotometer (Thermo FisherScientific Inc., USA). Briefly, 1 µg of total RNA per sample was used for reverse transcription (RT) using the Reverse Transcription Kit (TaKaRa, Japan) according to the manufacturer’s protocol. The SYBR Green I assay (TaKaRa, Japan) was used for quantitative PCR based on the manufacturer’s protocol. Briefly, 2.0 µl cDNA, 0.4 µl RT primer, 10.0 µl 2 × Master Mix, and 7.2 µl RNase free H_2_O were used in a 20 µl PCR reaction system on an Mx3005P (Agilent Technologies, USA). The results were analyzed using the 2^−ΔΔCT^ method. The data were analyzed using IBM SPSS statistic 19 software.

### Cell culture

The rat-derived cardiomyoblast cells, H9C2 cells^33^, were provided by the Center Laboratory of China-Japan Union Hospital and cultured in Dulbecco’s Modified Eagle Medium with Nutrient Mixture F-12 (DMEM/F12) supplemented with 10% fetal bovine serum (FBS) and 1% antibiotics in a modular incubator at 37 °C. At passage 3, cells were transferred to 6-well and 96-well plates in medium without antibiotics.

### Cell viability measurement

To imitate the process of HF *in vitro*, Ang II was used to induce apoptosis in H9C2 cells. Briefly, H9C2 cells were cultured in 96-well plates and treated with Ang II (0–100 nM) for 24 h, followed by cell viability determination with MTS using the CellTiter 96^®^ AQueous One Solution Cell Proliferation Assay (Promega, USA). Briefly, 100 µl medium with 20 µl MTS was mixed in each well. After incubation for 4 h, the absorbance at 490 nm was measured by ELISA to evaluate the cell survival rate.

### Transfection and Ang II stimulation

H9C2 cells were transfected with 3 µg fluorescently labeled miRNA mimics, inhibitor, or negative control (NC) using 7.5 µl Fugene HD (Promega, USA). After a transfection period of 6–24 h, the transfection efficiency was evaluated by fluorescence microscopy. Ang II was used to induce apoptosis in cells transfected with the above mentioned reagents. Briefly, prior to Ang II stimulation, culture media were removed, and cells were washed with phosphate-buffered saline (PBS) three times. After then, new media and Ang II at the specific concentration indicated were added. At 24 h post-Ang II stimulation, the cells were harvested for mRNA, microRNA, and Western blot analyses.

### Prediction of target gene of miR-199a-5p and primer design

The target genes for miR-199a-5p were predicted using bioinformatic tools Targetscan, mirbase, and pictar, and the specific primers for these genes were designed by Primer 5.0.

### Total protein isolation and Western blot

Protein samples were prepared from H9C2 cells with radioimmunoprecipitation assay (RIPA) lysis buffer, and the protein concentration was measured by the BCA protein assay kit. Briefly, 40 µg protein was separated by sodium dodecyl sulfate (SDS)-polyacrylamide gel electrophoresis (PAGE) and transferred to a polyvinylidene difluoride (PVDF) membrane, followed by blocking with 5% skim milk (BD Science, USA) or 5% BSA (Sigma-Aldrich, USA) in TBST (0.1% Tween 20 in Tris-buffered saline; 137 mmol/L NaCl and 20 mmol/L Tris/HCl, pH 7.4) for 1 h at room temperature. Membranes were then incubated overnight at 4 °C with the primary antibodies (Abcam, USA). Thereafter, membranes were washed with TBST and further incubated with the suitable horseradish peroxidase (HRP)-conjugated secondary antibody at room temperature for 1 h. The specific protein band was detected by using an ImageQuant LAS 4000 mini (GE Healthcare Bio-Sciences AB, USA) or a SuperSignal West Pico Chemiluminescence Kit (Thermo Fisher Scientific, USA). The intensities of the protein bands were quantified using ImageJ software (NIH).

### Caspase-3 activity measurement

Caspase-3 is a crucial member of the cysteine-requiring protease (caspase) family, and its activation is an established indicator of the apoptotic process^34^. Caspase-3 activity was measured with a Caspase-3 Activity Assay kit (Bestbio, China) according to manufacturer’s protocol. Briefly, 50 µg protein was added to 90 µl Detection Buffer and 10 µl Ac-DEVD-pNA for incubation at 37 °C for 1 h, followed by reading of the absorbance at A405 nm. The ratio of caspase-3 activity in the experimental group to that in the control group represented the relative caspase-3 activity.

### Flow cytometry

Following treatment, the cells were digested with trypsin, washed once with cold PBS, and then resuspended in 100 µl 1× binding buffer at a concentration of 1 × 10^5^ cells/ml. They were subsequently incubated with 5 µl annexin V fluorescein isothiocyanate and 5 µl propidium iodide (Annexin V:FITC Apoptosis Detection kit I, BD Biosciences, San Diego, CA, USA) for 20 min at room temperature. Thereafter, 400 µl of 1 × binding buffer was added to each tube, and the cell suspensions were analyzed by flow cytometry (BD LSRFortessa X-20, BD Biosciences, USA). The results were analyzed using FlowJo 7.61 software (Treestar Inc., Ashland, OR, USA)^35^.

### Luciferase reporter assay

To verify that miR-199a-5p is a direct target for miR-199a-5p, we constructed a target reporter using a pmiR-RB-REPORT™ (Ribobio, USA) carrier. We obtained an approximately 200-bp fragment of the 3′UTR of the target gene mRNA by gene synthesis (GENEWIZ, USA), in which two restriction sites, Not I (GC^GGCCGC) and Xho I (C^TCGAG), were inserted at the 3′ and 5′ termini, respectively. Next, the DNA fragment was ligated into the multiple cloning sites of pmiR-RB-REPORT™ (designed as pmiR-RB-REPORT™-WT). Targeted mutagenesis was performed to generate a specific mutation on the potential miR-199a-5p target site (from wt, CACACACTGGACTCTGGCCTGC, to mutant ACCATGTAGCACTCTG), designed as pmiR-RB-REPORT™ -mut. H9C2 cells seeded in 24-well plates were co-transfected with pmiR-RB-REPORT™-JunB-WT or pmiR-RB-REPORT™-JunB-mut and miR-199a-5p mimics or negative control (NC) duplex (GenePharma, USA) using FuGene HD transfection reagent. pmiR-RB-REPORT™ was transfected as a control. After 48 h, cells were harvested and luciferase activity was measured using a dual-luciferase reporter assay kit (Promega Corp, USA) and recorded by a multi-plate reader (Synergy 2, BioTek, USA).

### Rat Apoptosis RT² Profiler™ PCR Array analysis

The total RNA samples isolated as mentioned above were used for RT² Profiler™ PCR Array analysis. In brief, real-time PCR was performed with the RT^2^ SYBR Green qPCR Master Mix and the platform Rat Apoptosis RT^2^ Profiler™ PCR Array (Qiagen, Germany), the latter of which consisted of 84 profiles of the expression of 84 key genes, including positive/negative regulators of apoptosis- related genes. In this study, housekeeping genes such as β-actin and β-2 microglobulin were utilized to lower the variation in threshold values (CT). The values of controls in the plate were used to determine the expression of each target gene present in the plate. Equal aliquots of cDNA and RT^2^ SYBR Green qPCR Master Mix (25 μl) were added to each well of the same PCR Array plate containing the gene-specific primer sets. The conditions of the PCR program consisted of two cycling steps, with an initial cycle at 95 °C for 10 min, followed by 40 cycles of 15 s at 95 °C and 1 min at 60 °C. Upon completion of the qRT-PCR, the baseline and CT values were automatically set for each plate, and the same thresholds for all PCR arrays plates were kept. The data with the threshold values were exported and analyzed using the RT² Profiler PCR Array Data Analysis template v 3.3 (SuperArray Biosciences, USA).

### Construction of pBI-CMV3-JunB vector and transfection

To identify the effect of JunB on cell apoptosis, a JunB CDS sequence (Sango Biotech company, China) was subcloned into the pBI-CMV3 vector (Clontech, USA), which is a mammalian bidirectional expression vector constitutively expressing the protein of interest and ZsGreen1. The vector was transfected into H9c2 cells using Fugene reagent as described before. The transfection efficiency was evaluated by fluorescence microscopy at 24 h post-transfection. Western blotting and real-time PCR were performed to test whether transfection was successful.

### Flow cytometry and caspase-3 activity measurement

Flow cytometry and caspase-3 activity assays were performed as described above to evaluate the effect of JunB on H9c2 cell apoptosis.

### Statistical analysis

Data are presented as mean ± standard deviation (SD) and were compared using a two-tailed t-test for two groups or one-way analysis of variance (ANOVA) for experiments with more than two subgroups. Correlation analysis was performed by using Spearman correlation coefficient. P < 0.05 was considered statistically significant.
